# Investigation of structural, electronic and optical properties of two-dimensional MoS_2_-doped-V_2_O_5_ composites for photocatalytic application: a density functional theory study

**DOI:** 10.1098/rsos.230503

**Published:** 2023-07-19

**Authors:** Muhammad Hasnain Jameel, Muhammad Sufi bin Roslan, Mohd Zul Hilmi Bin Mayzan, Mohd Arif Bin Agam, Zaki I. Zaki, Ahmed M. Fallatah

**Affiliations:** ^1^ Department of Physics and Chemistry, Faculty of Applied and Technology (FAST), Universiti Tun Hussein Onn Malaysia, 84600 Muar, Johor, Malaysia; ^2^ Department of Chemistry, College of Science, Taif University, PO Box 11099, Taif 21944, Saudi Arabia

**Keywords:** two-dimensional MoS_2_-doped-V_2_O_5_ composite, MoS_2_ doping, optical conductivity, photocatalytic application

## Abstract

In the present research, the structural, electronic and optical properties of transition metal dichalcogenide-doped transition metal oxides MoS_2_-doped-V_2_O_5_ with various doping concentrations (*x* = 1–3%) of MoS_2_ atoms are studied by using first principles calculation. The generalized gradient approximation Perdew–Burke–Ernzerhof simulation approach is used to investigate the energy bandgap (*E_g_*) of orthorhombic structures. We examined the energy bandgap (*E_g_*) decrement from 2.76 to 1.30 eV with various doping (*x* = 1–3%) of molybdenum disulfide (MoS_2_) atoms. The bandgap nature shows that the material is a well-known direct bandgap semiconductor. MoS_2_ doping (*x* = 1–3%) atoms in pentoxide (V_2_O_5_) creates the extra gamma active states which contribute to the formation of conduction and valance bands. MoS_2_-doped-V_2_O_5_ composite is a proficient photocatalyst, has a large surface area for absorption of light, decreases the electron-hole pairs recombination rate and increases the charge transport. A comprehensive study of optical conductivity reveals that strong peaks of MoS_2_-doped-V_2_O_5_ increase in ultraviolet spectrum region with small shifts at larger energy bands through increment doping *x* = 1–3% atoms of MoS_2_. A significant decrement was found in the reflectivity due to the decrement in the bandgap with doping. The optical properties significantly increased by the decrement of bandgap (*E_g_*). Two-dimensional MoS_2_-doped-V_2_O_5_ composite has high energy absorption, optical conductivity and refractive index, and is an appropriate material for photocatalytic applications.

## 1. Introduction

Two-dimensional transition metal oxides (TMOs) and transition metal dichalcogenide (TMDs) composites are advanced and novel nanocomposites due to their unique optical, physical and chemical properties, that is dependent on the size as smaller nanoparticles are exhibiting quantum nature [1,2]. The excessive extinction coefficient, tune capacity, shape and dimension make them fairly traumatic in many fields, e.g. digital devices, nano-medicine and photocatalytic applications [3]. The chemical composition of MoS_2_, the valance shell of ‘S’ with −2 which is comfortable for oxidation, and C-S bonding atoms increase the stability of nanocomposite [4]. To boost the photocatalytic application of semiconductor photocatalysts, there are three foremost parameters that should be followed. Firstly, the increases of excitation wavelength, secondly decreases of electron-hole pair recombination, and lastly third very major point the increment of active sites around the surface edges for absorbance of the photon of light. The surface area for absorbance of light can be increased by following the strategies: firstly by inclusion with cations or anions, secondly by doping with metals element and at third factor by improving the structure nature of semiconductor photocatalysts to increase their surface area or porosity to enhance the photocatalytic application [5]. The MoS_2_ and V_2_O_5_ composite enrich the family with a large surface area with increased active sites around the surface edges for absorbance of the photon of light and decrease the recombination rate of electron-hole pair. MoS_2_-doped-V_2_O_5_ (V/MOS) composite is not only used as a two-dimensional photocatalyst, but also in sensors [6], electrochemistry [7], as a photo-anode in the sensitized solar cell [8], batteries [9] and photocatalytic application [10].

Vanadium is a rare earth element and it has a diversity of valance states. Among the family of oxides, V_2_O_5_ (vanadium pentoxide) has a proficient stable oxide structure and is mostly used in electrochemical instruments [11], thermoelectric devices [12], gas sensors [13], two-dimensional circuit board [14], oxidization accelerators and catalysis applications [15]. V_2_O_5_ is a prominent TMO belonging to group 5, d-block with unique properties such as 20 K boiling points and 936 K melting points. In the proficient advanced oxidation photocatalyst progression, the first step is electron-hole pair (eh*v*) creation during separation. These produced electron-hole pairs move to the V/MOS semiconductor surface and increase the oxidation and reduction of absorbed photons separately [16]. The lifetime of electrons and hole generation influence redox reaction [17]. The hole (h*v*) comprises sufficient to oxidize various organic pollutants, furthermore, $(O\dot{H})\;$ hydroxyl group in the water system. The hole can oxidize through various organic pollutants by creating $(O\dot{H})\;$ in water; moreover, the electrons in the conduction band interrelate with the oxygen of water $\left(H_{2}O\right)$ and create superoxide radicals that further interact with OH^−^ ion to form $(O\dot{H})\;$ radical. This $(O\dot{H})\;$ is a strong oxidizing candidate to the pollutant through oxidation of the organic molecule [18].

Two-dimensional V/MOS architectures are also greatly attractive in the field of energy harvesting and the environment. Photocatalysis is a proficient method for photo-anode, dye degradation, wastewater purification and water splitting [19]. Two-dimensional TMDs-doped-TMOs play a momentous role in the degradation of pharmaceuticals in industrial wastewater due to their low energy bandgap, less toxicity, best dispersibility etc. Nowadays, the discarding of pharmaceutical devastate has become a important concern worldwide. Separating various medications from wastewater effluents is often unsuccessful with convectional wastewater and biological treatments. However, pharmaceutical substances such as antibiotics, hormones, steroids and other drugs can neither be separated from effluent nor be destroyed by biological treatment [20]. As a consequence, heterogeneous photocatalysis has boosted as a promising technique for reducing the adverse effects of industrial consumption. Sunlight-active materials used as catalysts for destroying such pollutants are a realistic choice [21–23].

MoS_2_ is loaded on V_2_O_5_, whether the utmost valance state of V^+5^ and easily oxidized S^2−^ can hasten to electron during the reaction. There is no theoretical and experimental research on the structural electronic and optical behaviour of two-dimensional MoS_2_-doped-V_2_O_5_ with doping *x* = 1–3% atoms of MoS_2_ in the available literature. As a result, the primary aim of the present work is to provide some significant authentic information to the presented data on electronic, structural and optical properties of MoS_2_-doped-V_2_O_5_ using density functional theory through Cambridge Serial Total Energy Package (CASTEP) software simulation. MoS_2_-doped-V_2_O_5_ have high optical conductivity and are more consistent and appropriate surface area materials for photocatalytic applications. Two-dimensional MoS_2_-doped-V_2_O_5_ is used to determine appropriate and efficient photocatalytic applications. The results indicate that there is a decrement trend in the energy bandgap from 2.77 to 0.0 eV with doping *x* = 1–3% atoms of MoS_2_. MoS_2_-doped-V_2_O_5_ has high optical conductivity, is more consistent and has an appropriate surface area; it enhances photocatalytic activity. MoS_2_-doped-V_2_O_5_ has high optical conductivity as well as absorbance, is more consistent and is appropriate material for photocatalytic applications.

## 2. Computational methodology

The crystal structure of TMO (V_2_O_5_) pentoxide material with space group Fdd_2_ (no. 43) was used in the CASTEP program; moreover, before the analysis of the characteristics the geometry was optimized. CASTEP software, based on density functional theory (DFT), was executed on the accomplishment of our first-principles calculations. The bandgap and electronic exchange correlation have been deliberated by using generalized gradient approximation (GGA) and Perdew–Burke–Ernzerhof (PBE) parametrization method. The ultra-soft pseudo-potential was used to investigate the electrostatic interaction between ionic core and valance electrons. The atomic electronic configuration of vanadium (V_2_) and oxygen (O_5_) are $1s^{2}2s^{2}2p^{6}3s^{2}3p^{6}3d^{3}4s^{2}$ and $1s^{2}2s^{2}2p^{4}$, respectively. The novel ionic states creation is the result of nuclei interaction with the internal core and outer side electron. The outermost electrons in the MoS_2_-doped-V_2_O_5_ composite interact with the core electron, then the ions-electron potential converges. Brillouin zone integration is simulated through customized K-points; furthermore, these K-points meshes build by 1 × 1 × 1 on the Monkhorst pack grid for optimization of the crystal structure. During the complete optimization, the total energy keeps constant at 1 × 10^−5^ eV. Moreover, the convergence assessment of applied forces to the MoS_2_ atoms is set aside at −2.283 eV/Å. The geometry is optimized through the CASTEP algorithm with doping *x* = 1–3% atoms of MoS_2_ in V_2_O_5_.

## 3. Results and discussion

### 3.1. Structural analysis

**Figure 1.**
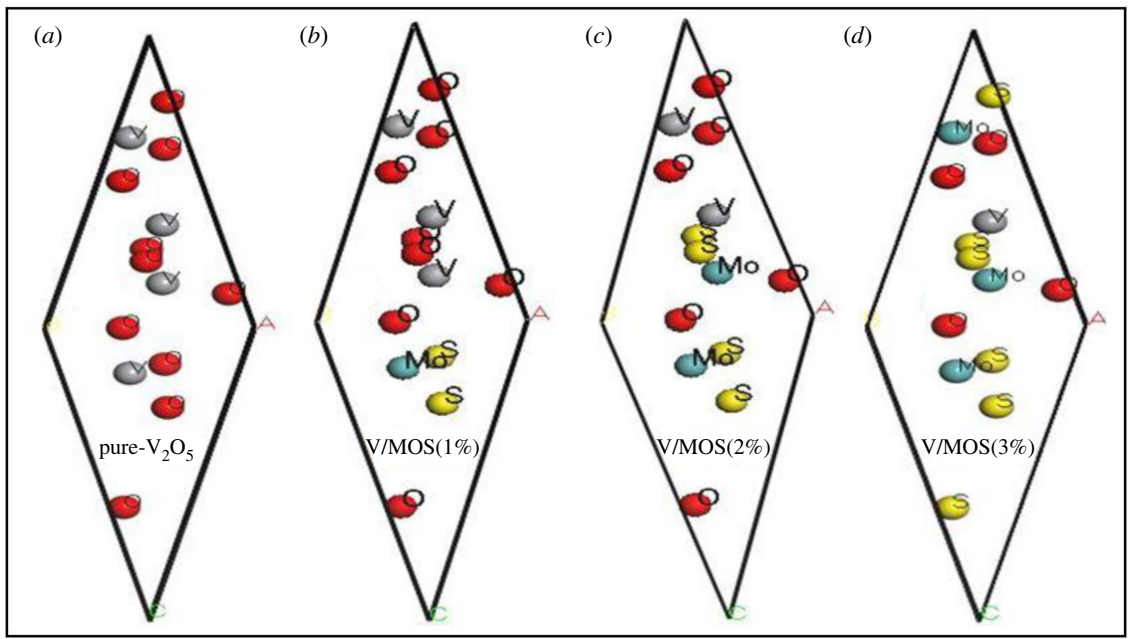
Supercell cell structure of two-dimensional (TMDs-doped-TMOs) MoS_2_-doped-V_2_O_5_ composite (*x* = 1%, 2% and 3%).

The supercell 1 × 1 × 1 of MoS_2_-doped-V_2_O_5_ is shown in figure 1. The orthorhombic structure of MoS_2_-doped-V_2_O_5_ with space group Fdd2 (no. 43) [24–28]. The crystal lattice parameters of two-dimensional MoS_2_-doped-V_2_O_5_ is oriented through *a* = 11.044, *b* = 10.315, *c* = 5.700 and angles *α* = 81.723°, *β* = 67.562°, *γ* = 30.715° are shown in table 1. The overall energy of MoS_2_-doped-V_2_O_5_ crystal is maintained low level by estimating equilibrium lattice parameters with the help of the Monkhorst pack grid, and results are obtained throughout a wide range of lattice parameter values. The decrement in lattice parameters values with various doping (*x* = 1–3%) atoms of molybdenum disulfide (MoS_2_) as a result there is presented a strong attraction between MoS_2_ and V_2_O_5_ atoms. Furthermore, doping with (*x* = 1–3%) MoS_2_ atoms, the decrement in supercell volume varies from 282.25 to 123.71 Å as shown in table 2. This indicates super-cells of MoS_2_-doped-V_2_O_5_ are remarkably shrunk as a result of decrement in the energy bandgap (*E_g_*).

**Table 1.** The lattice constants/angles of the supercell of MoS_2_-doped-V_2_O_5_.

material	lattice constant Å/ angles			binding energy (eV/atom)
pure V_2_O_5_	*a* = 11.044	*b* = 10.315	*c* = 5.700	−2.283 eV
angles	*α* = 81.723°	*β* = 67.562°	*γ* = 30.715°	
space group	Fdd2 (no. 43)

**Table 2.** The energy bandgap (*E_g_*) and volume of super cells of two-dimensional MoS_2_-doped-V_2_O_5_ composite (*x* = 1%, 2% and 3%).

compound	volume (A)^3^	bandgap (eV)
pure V_2_O_5_	282.25	2.76
MoS_2_-doped-V_2_O_5_ composite (*x* = 1%)	201.71	2.20
MoS_2_-doped-V_2_O_5_ composite (*x* = 2%)	140.12	1.76
MoS_2_-doped-V_2_O_5_ composite (*x* = 3%)	123.71	1.30

### 3.2. Electronic band structures and density of states

The band structure of a crystal is relating to energy eigenvalues and plays a vital role to understand energy Fermi (*E_F_*) levels which determine whether the composite is conductor, semi-conductor or a insulator. There are two types of bands first one is conduction band (CB) which is located at the accurately above the energy fermi level (*E_F_*) as well as valance band (VB) which is located below the *E_F_*.

**Figure 2.**
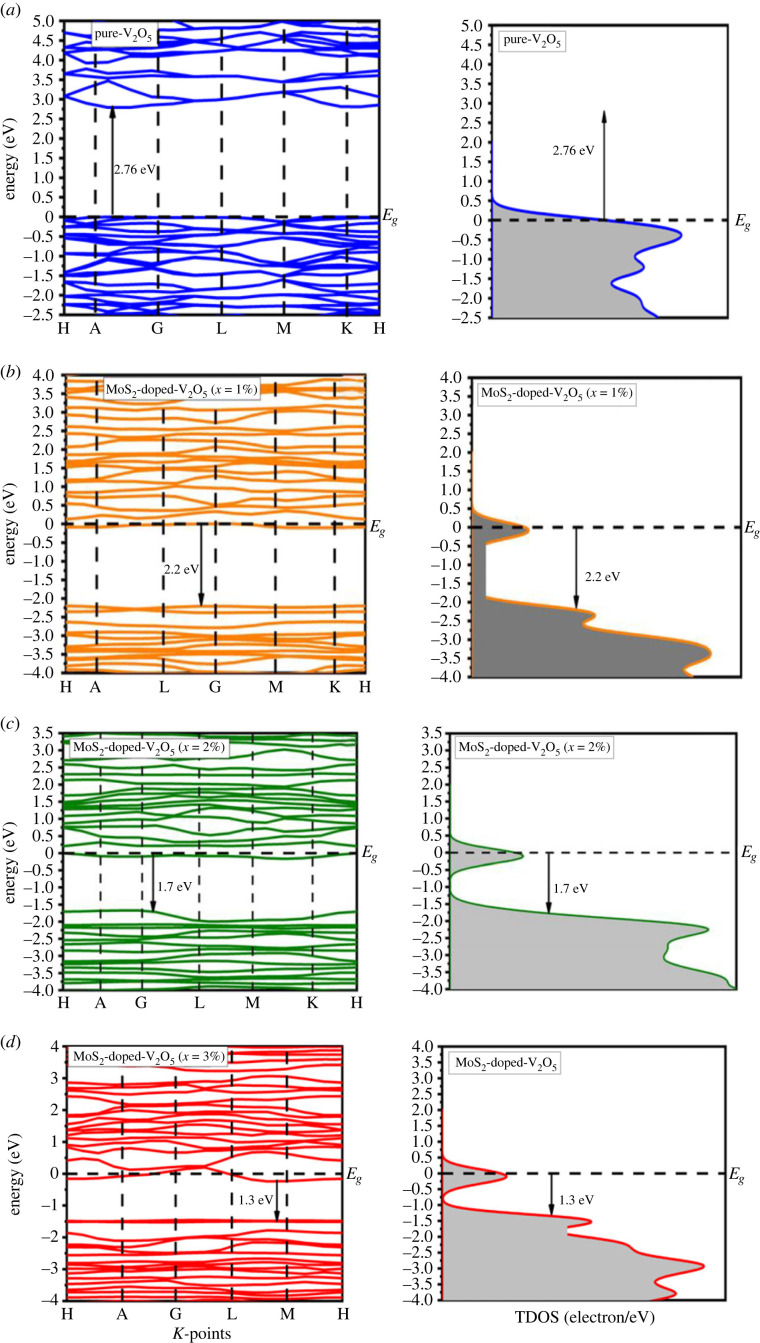
(*a–d*) Band structures of two-dimensional (TMDs-doped-TMOs) MoS_2_-doped-V_2_O_5_ composite (*x* = 1%, 2% and 3%).

**Figure 3.**
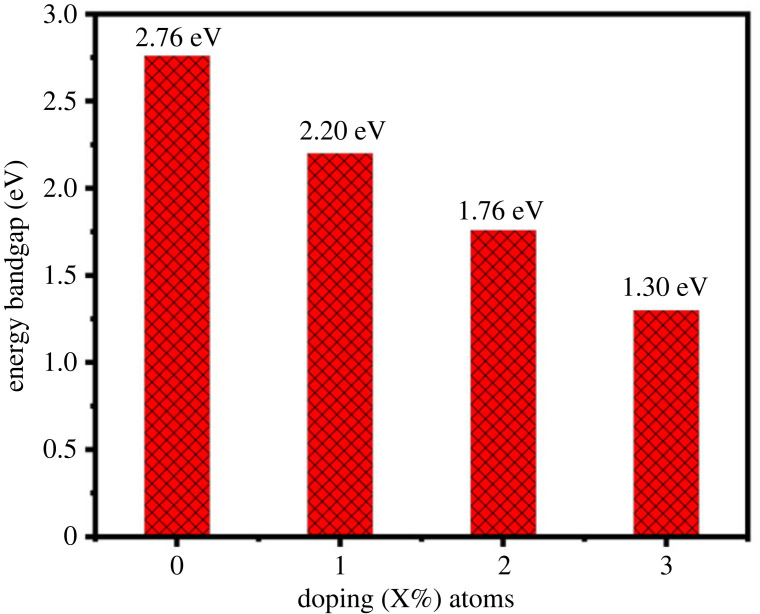
Bandgap doping concentration (*X*%) atoms of two-dimensional (TMDs-doped-TMOs) MoS_2_-doped-V_2_O_5_ composite.

Figure 2 shows that the upper position of valance band maxima (VBM) and lower position of conduction band minima (CBM) are accurately positioned at the same point ‘G’ represents that MoS_2_-doped-V_2_O_5_ eminent direct semiconductor. In the current study, the direct bandgap is reported with various doping (*x* = 1–3%) atoms of MoS_2_. The band structures of two-dimensional MoS_2_-doped-V_2_O_5_ composite are shown in figure 2*a–d*; these are obtained by doping (*x* = 1–3%) atoms of MoS_2_. The minimum bandgap of 1.3 eV is obtained at 3% MoS_2_ atoms. A decrement trend in the MoS_2_-doped-V_2_O_5_ band structures can be examined through increased doping *x* = 1–3% atoms as shown in figure 3. The bandgap values and volume of super cells are decreasing as shown in table 2. Due to a decrease in the volume of super-cells with increased doping atoms (*x* = 1–3%) of MoS_2_ in V_2_O_5,_ the CB moves toward the Fermi level (*E_F_*) along the G symmetry, which is the main reason for the reduction of the energy bandgap (*E_g_*) of the MoS_2_-doped-V_2_O_5_ materials. The bandgap structures revealed that two-dimensional material appears in semiconductors within direct bandgap nature. These findings show that two-dimensional MoS_2_-doped-V_2_O_5_ is a suitable material for photocatalytic applications.

**Figure 4.**
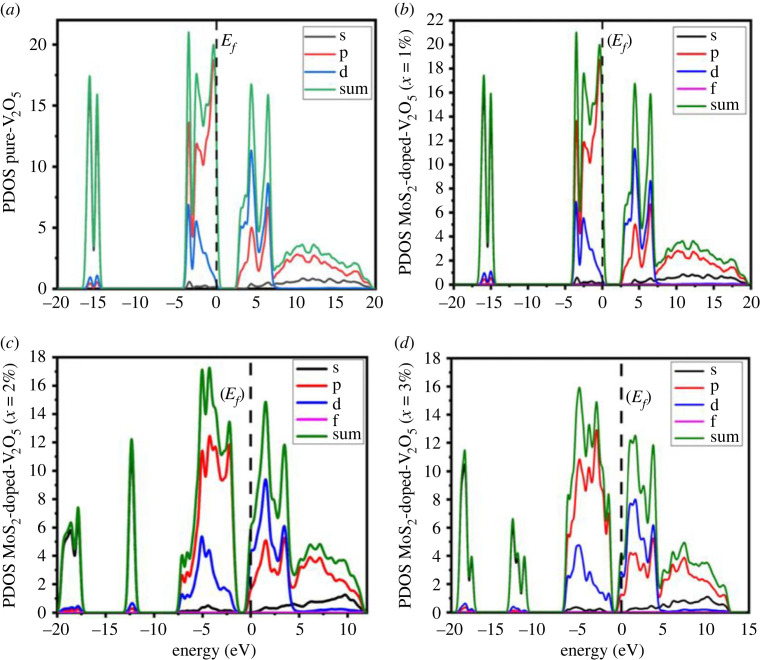
(*a–d*) PDOS and TDOS of two-dimensional (TMDs-doped-TMOs) MoS_2_-doped-V_2_O_5_ composite (*x* = 1%, 2% and 3%).

The electronic bandgap represented per unit energy is measured through total density of states although ions' contribution of various band structures is analysed by partial density of states. The total density of states (TDOS) and partial density of states (PDOS) are used to explicate the decrement in the bandgap. The Fermi level $\left(E_{f}\right)$, which is indicated by a dotted line, is set at the peak of the valence band. Figure 4*a*–*d* shows with increased doping (*x* = 1–3% atoms), its value tends to decrease from 20 to the minimum value of 16 at 3%. This characteristic peak shifts towards the right through the doping concentration of 3%. Moreover, in p states, the number of gamma states increases as the doping atoms increase. As a consequence, p states represent more major contributions than s and d states, as shown in figure 4*a*–*d*. By increasing doping (*x* = 1–3%) atoms, the hybridization of p-d states increases as a result of decrement in an energy bandgap (*E_g_*). These results indicate that the MoS_2_-doped-V_2_O_5_ composite is a suitable material for photocatalytic applications.

When through increased *x* = 1–3% atoms the VB moves near a lower energy level, although the CB shifts towards the lower energy, which is the basic motive for the decrement of the bandgap. With the help of all observations, we can conclude that the PDOS shifts towards minimum energies as a consequence of doping *x* = 1–3% atoms, leading to a decrement in the bandgap, which also implies the optical properties described below.

### 3.3. Optical properties

The optical properties of (two-dimensional) TMOs and TMDs composites are fascinating and have noteworthy photocatalytic applications. The complex ε(ω) dielectric parameters explain the response of two-dimensional MoS_2_-doped-V_2_O_5_ composite (*x* = 1%, 2% and 3%) to an electric field. It is divided into two components, real dielectric function (RDF) and imaginary dielectric function (IDF), and is dependent on the crystal's optical band structure.

The optical properties of two-dimensional MoS_2_-doped-V_2_O_5_ composite may be described with the help of electronic structure moreover relative permittivity, refractive index, coefficient of absorption, reflectivity as well as energy loss function. These properties are very helpful in signifying the viability and suitability of two-dimensional structured MoS_2_-doped-V_2_O_5_ material in photocatalytic applications. All of the optical conductivity properties are the result of the interaction of valance electrons among core inner electrons of MoS_2_-doped-V_2_O_5_ composite and waves (electromagnetic waves). All these properties are connected, and the complex dielectric function can be used which is given in [25,29,30].

3.1 $$\varepsilon (\omega )=(\varepsilon_{1}\omega )+i\varepsilon_{2}(\omega ).$$

To study the optical response to the effect of doping (*x* = 1–3%) atoms of MoS_2_ on RDF $\left(\varepsilon_{1}\omega \right)$ and IDF $\varepsilon_{2}(\omega )$ of the dielectric function ε(ω) can be measured through the following equations (3.2) and (3.3) [31,32]:

3.2 $$\varepsilon_{2}(\omega )=\;-\frac{Ve^{2}}{2\pi \hbar m^{2}\omega^{2}}\int d^{3}k\underset{nn^{\prime }}{\sum }1<kn|P|k\tilde{n}>I^{2}f(k)\times (1-f(k\tilde{n})\delta (E_{kn}-E_{k\tilde{n}\;}-\hbar \omega ),$$

3.3 $$(\varepsilon_{1}\omega )=1+\frac{2}{\pi }P\overset{\infty }{\underset{0}{\int }}\frac{\omega^{\prime }\varepsilon_{2}(\omega^{\prime })d\omega^{\prime }}{{\omega^{\prime }}^{2}-\omega^{\prime }}=n^{2}(\omega )-k^{2}(\omega ).$$

**Figure 5.**
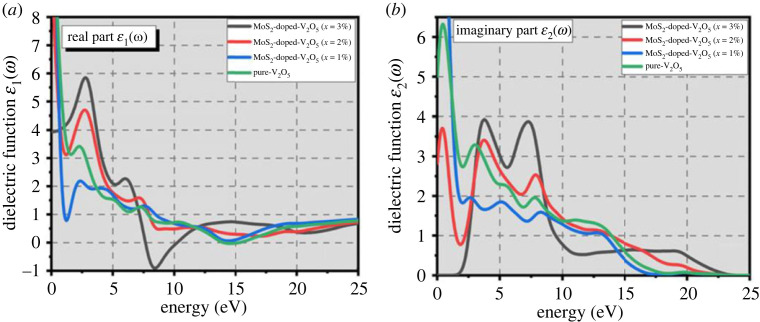
Dielectric functions (*a*) RDF and (*b*) IDF of two-dimensional (TMDs-doped-TMOs) MoS_2_-doped-V_2_O_5_ composite (*x* = 1%, 2% and 3%).

The dielectric constants RDF $\left(\varepsilon_{1}\omega \right)$ and IDF $\varepsilon_{2}(\omega )$ present the relative permittivity of the two-dimensional MoS_2_-doped-V_2_O_5_ composite. If we take the term dielectric literally, it shows how much an electric field is permitted to go through MoS_2_-doped-V_2_O_5_ composite at different doping *x* = 1–3% atoms. This mostly exhibits how much polarization MoS_2_-doped-V_2_O_5_ composite can be tolerated at doping atoms. As a result, an ideal electrical conductor should have zero value because no field can be there within conductor boundaries. The RDF $\left(\varepsilon_{1}\omega \right)$ gives information regarding the polarization intensity of MoS_2_-doped-V_2_O_5_ at different percentages of doping atoms. The main peak of dielectric function at 1% atom at 3.2 eV and at 3% it reaches 5.8 eV as shown in figure 5*a,b*. It seems to decrease towards a high value of 7.5 eV when with various doping (*x* = 1–3% atoms) of MoS_2_, respectively. The optical characteristics are interlinked to energy dissipation and are affected by IDF $\varepsilon_{2}(\omega )$ function. The IDF is shown in figure 5*b*. As doping (*x* = 1–3%) atoms of MoS_2_ increased, IDF peaks shift slightly to the lower value, and energy also shifted towards higher values. All peaks slightly move toward higher energy when doping increases. These dielectric results show that two-dimensional MoS_2_-doped-V_2_O_5_ is a suitable material for photocatalytic applications.

### 3.4. Refractive index and extinction

**Figure 6.**
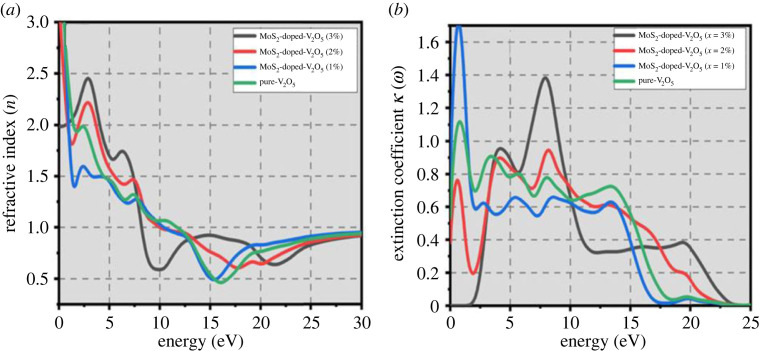
(*a*) Refractive index (*n*) and (*b*) extinction coefficient of two-dimensional (TMDs-doped-TMOs) MoS_2_-doped-V_2_O_5_ composite (*x* = 1%, 2% and 3%).

By vigilant investigation of the extinction coefficient *k*(*w*) and refractive index *n*(*w*) of two-dimensional MoS_2_-doped-V_2_O_5_ transition material the absorbance of electromagnetic (EM) radiation and the calculation of optical transparency is shown in figure 6*a*,*b*. The behaviour of refractive index *n*(*w*) and extinction coefficient *k*(*w*) in the range of 0–50 eV with doping (*x* = 1–3% atoms) of MoS_2_ of two-dimensional MoS_2_-doped-V_2_O_5_ composite.

At 0 eV two-dimensional MoS_2_-doped-V_2_O_5_ has a refractive index (*n*) of 1.5, 2, 2.3 and 2.5 with pure and doping (*x* = 1–3% atoms) of MoS_2_, respectively. The refractive move toward decreasing radically with energy eV up to 5 eV. The refractive index (*n*) fluctuates from 0 to 5 in the range of energy 0 to 50 eV outstanding to the diverse frequencies of the inner-transition band. The lower refractive index shows smaller polarization in the eminent energy range. The following equations are used to calculate the additional optical factors such as reflectivity, absorption, refractive index and energy loss [15,33–36]:

3.4 $$a(\omega )=\frac{4k\pi }{\lambda }=\frac{\omega }{nc}\varepsilon_{2}(\omega ),$$

3.5 $$a(\omega )=2\omega k(\omega )=\sqrt{2}\;{\left[{\{\varepsilon_{1\;}{\left(\omega \right)}^{2}+\varepsilon_{2}{\left(\omega \right)}^{2}\}}^{1/2}-\varepsilon_{1}\left(\omega \right)\right]}^{1/2},$$

3.6 $$L(\omega )=\;\frac{\varepsilon_{2}}{\varepsilon_{1}{\left(\omega \right)}^{2}+\;\varepsilon_{2}{\left(+\right)}^{2}},$$

3.7 $$I(\omega )=\sqrt{2}\omega {\left(\sqrt{\varepsilon_{1}{\left(\omega \right)}^{2}}\;+\;\sqrt{\varepsilon_{2}{\left(\omega \right)}^{2}}-\varepsilon_{1}(\omega )\right)}^{1/2},$$

3.8 $$n(\omega )=\;\left(\frac{1}{\sqrt{2}}\right){\left(\sqrt{\varepsilon_{1}{\left(\omega \right)}^{2}}+\;\sqrt{\varepsilon_{2}{\left(\omega \right)}^{2}}-\varepsilon_{1}(\omega )\right)}^{1/2},$$

3.9 $$K(\omega )=\frac{I(\omega )}{2\omega },$$

3.10 $$r(\omega )=\;\frac{n+iK-1}{n+iK+1},$$

3.11 $$\sqrt{\varepsilon (\omega )}=n(\omega )+iK(\omega )=N(\omega ),$$

3.12 $$\varepsilon_{1}(\omega )=\;n^{2}-K^{2},$$

3.13 $$\varepsilon_{2}(\omega )=2nK.$$

### 3.5. Absorbance and energy loss

**Figure 7.**
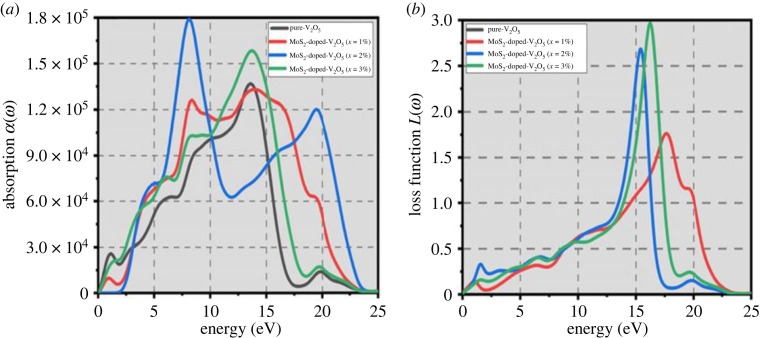
(*a*) Absorption and (*b*) energy loss function of two-dimensional (TMDs-doped-TMOs) MoS_2_-doped-V_2_O_5_ composite (*x* = 1%, 2% and 3%).

The absorption of any material is capably connected to the capability of absorbance luminescent EM radiation in disparity to photons with appropriate energy (E=ℏω), furthermore, the energy loss function L(ω) describes the energy dissipation when the material comes into range with incident photons as shown in figure 7*b*. The absorbance of two-dimensional MoS_2_-doped-V_2_O_5_ is increasing by doping (*x* = 1%, 2% and 3%) atoms of MoS_2_ as shown in figure 7*a*. The photon energy increase through increasing doping (*x* = 1%, 2% and 3%) atoms of MoS_2_, and the absorption coefficient of MoS_2_-doped-V_2_O_5_ rises, achieving 1.8 × 10^5^ cm^−1^ at 5 eV with 3%. The results indicate that two-dimensional MoS_2_-doped-V_2_O_5_ has high optical conductivity as well as absorbance and is a more consistent and appropriate material for photocatalytic applications.

### 3.6. Optical conductivity and reflectivity

**Figure 8.**
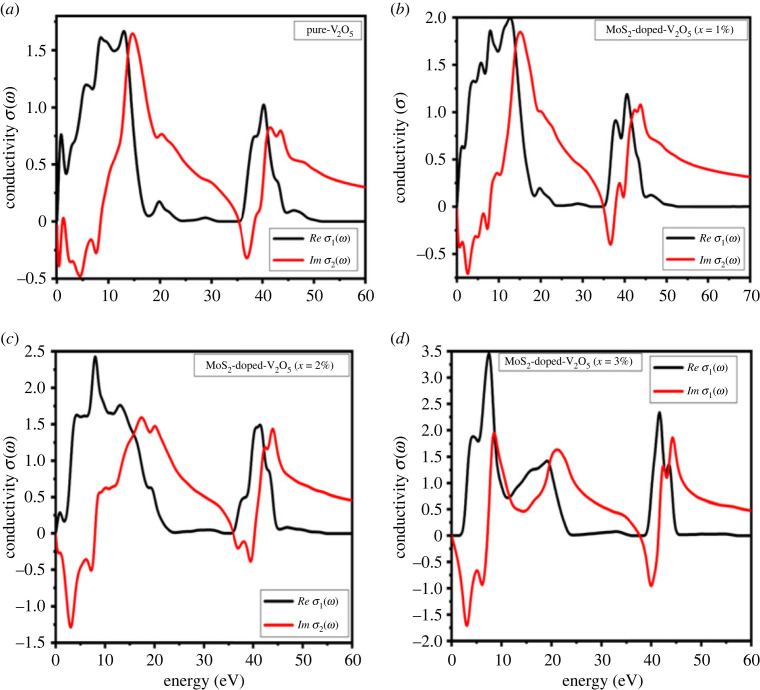
(*a*–*d*) Optical conductivity of two-dimensional (TMDs-doped-TMOs) MoS_2_-doped-V_2_O_5_ composite (*x* = 1%, 2% and 3%).

Optical conductivity describes the conductance of photogenerated electrons by the photoelectric phenomenon. Electromagnetic radiation EM is used to break the bonding of particles. Figure 8*a*–*d* shows the optical conductance of MoS_2_-doped-V_2_O_5_ composite in the range of 0 to 60 eV. The real peaks of optical conductance σ1(w) initiate from the origin point and touch its maximum conductivity values of 1.7, 2, 2.5 and 3.5 cm^−1^ at the energy range 0 to 20 eV with pure V_2_O_5_ and increasing doping (*x* = 1%, 2% and 3%) atoms of MoS_2_. The real optical conductivity of two-dimensional MoS_2_-doped-V_2_O_5_ composite initially increases from the starting zero point and at 5 eV and suddenly decreases till 30 eV.

On the other hand, imaginary optical conductivity σ2(w) shows the largest value at 5 eV: 1.7, 1.6, 1.4 and 1.6 cm^−1^ for pure V_2_O_5_ and doping (*x* = 1%, 2% and 3%) atoms of MoS_2_. The optical conductivity is increasing with the increased doping concentration of MoS_2_. These results of optical conductivity indicate that two-dimensional MoS_2_-doped-V_2_O_5_ is appropriate material for photocatalytic applications.

**Figure 9.**
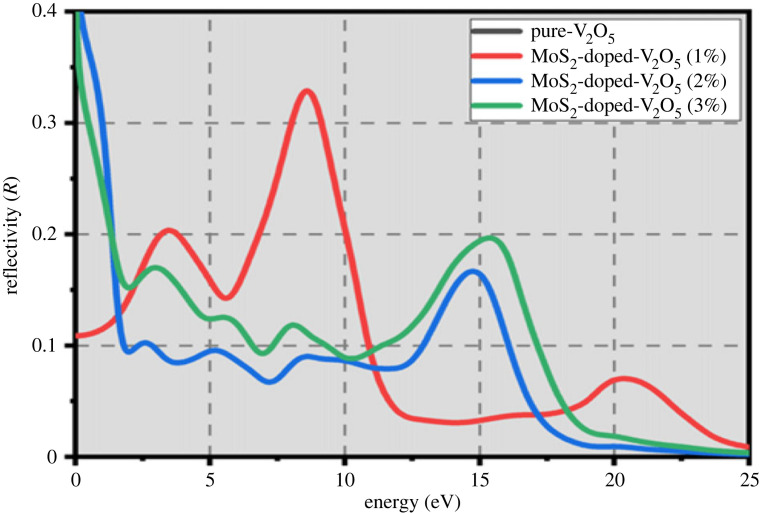
Reflectivity of two-dimensional (TMDs-doped-TMOs) MoS_2_-doped-V_2_O_5_ composite (*x* = 1%, 2% and 3%).

The reflectivity of any material can be used to examine its surface behaviour. The reflectivity surface behaviour of two-dimensional MoS_2_-doped-V_2_O_5_ composite at different doping (*x* = 1%, 2% and 3%) atoms of MoS_2_ is shown in figure 9. At 0 eV, the value of reflectivity for MoS_2_-doped-V_2_O_5_ material is 0 for pure V_2_O_5_, 0.32 at 1% atom, 0.15 at 2% atom and 0.7 at 3% atom, respectively. These values show that the reflectivity peak slightly shifts towards the higher values of frequency as doping concentration increases. These results of reflectivity indicate that two-dimensional MoS_2_-doped-V_2_O_5_ composite is a suitable material for photocatalytic applications.

### 3.7. Photocatalytic mechanism

**Figure 10.**
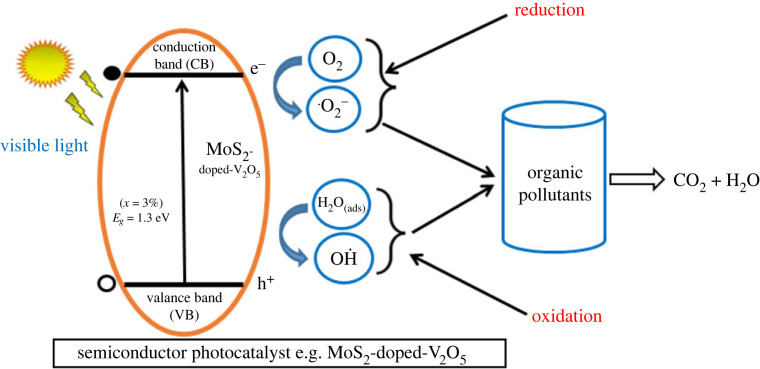
Schematic diagram of the photocatalytic mechanism of two-dimensional (TMDs-doped-TMOs) MoS_2_-doped-V_2_O_5_ composite (*x* = 1%, 2% and 3%) under sunlight.

Figure 10 schematic diagram shows that the sunlight conciliates photo-degradation application of two-dimensional MoS_2_-doped-V_2_O_5_ composite. Two-dimensional MoS_2_-doped-V_2_O_5_ composite with a large surface area absorbs the waste material more proficiently through active surface sites as equation (3.14). Under the sunlight, revelation two-dimensional (TMDs-doped-TMOs) MoS_2_-doped-V_2_O_5_ electrons and holes in the CB and VB correspondingly as equation (3.15). Organic particle becomes excited in the exposure of sunlight, and it moves from HOMO toward LUMO of pollutant level. Furthermore, electrons are moved from the VB to the CB of two-dimensional MoS_2_-doped-V_2_O_5_ and increase electron density with increased doping (*x* = 1%, 2% and 3% atoms). The photo-captured electrons are positioned in the conduction level of MoS_2_-doped-V_2_O_5_ renovating the oxygen molecule into super-oxide ${\;}^{\cdot }O_{2}^{\;-}$ radical as equation (3.16). At a similar moment, $O\dot{H}\;$hydroxyl radical produced the holes in the valance level through water splitting molecules as equations (3.17) and (3.18). Both superoxide and hydroxyl (unsaturated form) changed the pollutant into CO_2_ and H_2_O as equation (3.19). Underexposure of sunlight photocatalytic activity of organic waste pollutants by MoS_2_-doped-V_2_O_5_ is given below [37–39].

3.14 $$\begin{array}{rl} & \mathrm{dye}\;\mathrm{molecules}+{\mathrm{MoS}}_{2}-\mathrm{doped}-V_{2}O_{5}\to {\mathrm{MoS}}_{2}-\mathrm{doped}\\ & \;-V_{2}O_{5}+\mathrm{absorbed}\;\mathrm{dye}\;\mathrm{molecule}\;\mathrm{on}\;\mathrm{Mo}S_{2}(\mathrm{absorption}), \end{array}$$

3.15 $$H\nu +{\mathrm{MoS}}_{2}-\mathrm{doped}-V_{2}O_{5}\to \;{\mathrm{MoS}}_{2}-\mathrm{doped}-V_{2}O_{5}(h_{\mathrm{VB}}^{+}+e_{\mathrm{CB}}^{-}),$$

3.16 $$e_{\mathrm{CB}}^{-}+O_{2\;(\mathrm{ads})}\to {\dot{O}}_{2}^{-},$$

3.17 $$h_{\mathrm{VB}}^{+}+H_{2}O_{\left(\mathrm{ads}\right)}\to H^{+}+O\dot{H},$$

3.18 $$O\dot{H}+\mathrm{absorbed}\;\mathrm{dye}\to {\mathrm{CO}}_{2}+H_{2}O$$

3.19 $$\mathrm{and}{\;}^{\cdot }O_{2}^{-}+\mathrm{absorbed}\;\mathrm{dye}\to {\mathrm{CO}}_{2}+H_{2}O.$$

The computed results show that under visible light, active sites of the MoS_2_-doped-V_2_O_5_ composite may be proficient to make excellent photocatalytic activity. Two-dimensional (TMDs-doped-TMOs) MoS_2_-doped-V_2_O_5_ composite semi-conductor is appropriate material for photocatalytic applications.

## 4. Conclusion

In the present research, the structural, electronic and optical properties of TMDs-doped-TMOs MoS_2_-doped-V_2_O_5_ with various doping (*x* = 1–3% atoms) of MoS_2_ is studied by using first principles calculation. The GGA-PBE simulation approach is used to investigate orthorhombic structures. It is examined that the energy bandgap (*E_g_*) decrement from 2.76 to 1.3 eV with direct semiconductor nature through various doping (*x* = 1–3% atoms) of MoS_2_. MoS_2_ doping (*x* = 1–3% atoms) in pentoxide (V_2_O_5_) creates the extra gamma active states which contribute to the formation of conduction and valance bands. MoS_2_-doped-V_2_O_5_ is a proficient composite for photocatalysis, has a large surface area for light absorption, decreases the electron-hole pairs recombination rate and increases the charge transport. A comprehensive study of optical conductivity reveals that strong peaks of MoS_2_-doped-V_2_O_5_ increase in ultraviolet spectrum region with small shifts at larger energy bands through increment doping *x* = 1–3% atoms of MoS_2_. A significant decrement was found in the reflectivity due to the decrement in the bandgap with doping *x* = 1–3% atoms of MoS_2_. The optical properties significantly increased by the decrement of *E_g_*. Two-dimensional MoS_2_-doped-V_2_O_5_ composite has high energy absorption, optical conductivity, and refractive index and is an appropriate material for photocatalytic applications.

## Data Availability

The data are provided in the electronic supplementary material [40].
